# Analysis of pedestrian wayfinding under herd effect in VR fire evacuation at indoor library: gender difference considered

**DOI:** 10.3389/fpsyg.2025.1558115

**Published:** 2025-09-30

**Authors:** Jianping Li, Chuanjin Liu

**Affiliations:** ^1^Faculty of Education Science, Sichuan Normal University, Chengdu, China; ^2^Division of Humanities and Social Sciences, Sichuan Normal University, Chengdu, China; ^3^School of Marxism, Chengdu College of Arts and Sciences, Chengdu, China

**Keywords:** immersive virtual reality, pedestrian wayfinding behavior, fire emergency evacuation, crowd flow, herd effect, gender difference

## Abstract

**Background:**

As libraries are critical areas for fire safety and evacuation, there is a need to expand research on pedestrian evacuation in this scenario.

**Methods:**

We designed an immersive virtual reality (IVE) experiment to examine the wayfinding choices made by pedestrians during a library fire under conditions of different crowd patterns represented by non-game players and differences in gender ratios. A total of 162 participants were asked to engage in an evacuation task in a randomized order across sixteen different experimental scenarios.

**Results:**

(1) Under the influence of crowd patterns, pedestrians tended to follow the route chosen by the majority of the evacuating crowd. (2) Pedestrians tended to follow the group with a higher proportion of males in the evacuation. (3) When the proportion of males in the route chosen by the majority of the evacuating population is significantly smaller than the proportion of females, the pedestrian’s choice of that route is significantly lower. (4) The gender ratio significantly attenuates the influence of the herd effect on the subjects’ route decision-making.

**Conclusion:**

This experiment expands the study of pedestrian routing behavior in a fire situation and provides some empirical evidence for the further improvement of fire evacuation.

## 1 Introduction

Indoor fire accidents occur frequently, resulting in huge economic losses and even directly threatening the safety of pedestrians. In recent years, university libraries have been expanding in scale, with more functions and complex indoor structures; increased collection volume, more combustible materials and fast burning speed, and strong uncertainty in the direction of fire propagation, resulting in high fire loads (Zhang et al., 2024). Libraries are major on-campus population gathering places, which are critical areas for fire safety and evacuation, and pose a high threat to emergency pedestrian evacuation (Bahmani et al., 2023). Current indoor scenarios for pedestrian emergency wayfinding are mostly focused on residential buildings, shopping malls, and subways, and fewer studies have focused on college libraries (Lin et al., 2020a; Fu et al., 2021; Wang et al., 2022). Therefore, we designed a study on pedestrian wayfinding decisions during fire emergencies in university indoor libraries.

As the most unpredictable factor in fire evacuation, pedestrians are the key to reducing the degree of fire hazards (Zhang et al., 2023): on the one hand, if pedestrians can quickly and safely evacuate when a fire breaks out, it can minimize casualties, facilitate firefighters’ firefighting and search and rescue work, and reduce the chaos and danger in the rescue process; on the other hand, if the crowd runs blindly and conveys panic, it is very easy to cause road blockage and trampling accidents, and also increase the risk of asphyxiation due to prolonged exposure to smoke generated by fire (Moussaïd et al., 2016; Ríos and Pelechano, 2020; Lin et al., 2023). Therefore, an in-depth study of the wayfinding choice mechanisms of pedestrians would be beneficial to reduce the fire threat.

A large number of experiments have explored the factors influencing people’s wayfinding choices in fire situations, with social influence being shown to play a greater role. Social influence has been defined as a clustering phenomenon in which individuals often exhibit following others in evacuating together when making routing choices during an emergency (Lin et al., 2020b). Most experiments have been designed with crowds concentrating on a single route to evacuate and observing whether participants choose to follow or not; however, in actual evacuations, people do not concentrate on a fixed route and often show that pedestrians evacuate on all available routes, but there is inconsistency in the proportion of evacuees (Liu and Mao, 2022; Lin et al., 2023). Therefore, how different crowd flows affect pedestrian evacuation route selection needs to be further investigated.

In addition, it is worthwhile to explore in depth whether individual characteristics in evacuating crowds, especially the gender distribution ratio, lead to changes in pedestrians’ wayfinding decisions. According to social role orientation theory, research has shown that individuals are assigned specific roles in society and that these roles carry corresponding behavioral patterns and expectations (Lovreglio et al., 2016a). In fire emergencies, men are perceived to have greater psychological resilience and spatial awareness. They are more likely to be positioned as safe exit guides. In contrast, women are perceived to have better communication skills and emotional calming abilities and are more likely to be positioned as evacuation facilitators and active cooperators (Rosenthal et al., 2012; Mao et al., 2024). Therefore, our experiment also explored the effect of gender distribution ratio on pedestrians’ wayfinding decisions.

## 2 Literature review

### 2.1 Wayfinding behavior

Wayfinding is defined as the process by which pedestrians perceive information about their environment, recognize their localization, and find a path to reach a desired spatial destination. More specifically, wayfinding is designed to fulfill the pedestrian’s purpose of reaching a specific location (Cao et al., 2019). Perceiving the environment as the first step of wayfinding has an important impact on all subsequent decisions (Fu et al., 2023; Fu et al., 2024a), so it is necessary to investigate how environmental factors affect wayfinding pedestrian decisions. According to related studies, environmental attributes are mainly classified into static and dynamic factors. Static factors include the physical characteristics of the building (Natapov et al., 2022): the width and inclination of the stairs, the length of the corridor, and the angle of the corner; and the evacuation signs (Zhu et al., 2020): the location, color, shape, and slogans. Dynamic factors include fire physical factors (Lovreglio et al., 2022): flame location, smoke concentration, fire size; social influences (Chen et al., 2020): proportion of crowd route choice, crowd density, and proportion of gender distribution.

Fire emergency wayfinding is different from daily wayfinding, which requires pedestrians to bear greater psychological pressure and panic. Therefore, pedestrians in emergencies do not have sufficient time to weigh the pros and cons and synthesize information about the properties of the surrounding environment to make fully rational wayfinding decisions. It has been found that dynamic factors, which pedestrians more easily perceive, exert greater benefits on pedestrian decision-making (Xie et al., 2021). Social influence has received a great deal of research attention as one of the key dynamic factors influencing evacuation behavior in fire situations (Kinateder and Warren, 2021). Most of the current studies have demonstrated the role of the herd effect in influencing pedestrian evacuation wayfinding, i.e., individuals often exhibit a clustering phenomenon of following others to evacuate together during an emergency evacuation in a building (Tong and Bode, 2023; Fu et al., 2024b). However, little further consideration has been given to the specific effects of different crowd diversion ratios on pedestrians’ wayfinding decisions. In addition, whether the gender ratio of evacuating crowds affects pedestrians’ wayfinding choices needs to be further explored.

### 2.2 Virtual reality experiment

Virtual reality (VR) is a computer simulation system that allows the creation and experience of virtual worlds, utilizing computer technology, graphic and image technology, sensor technology, network technology, etc. to generate realistic three-dimensional virtual environments, which endow the user with feelings including visual, auditory, and tactile sensations to make the user feel immersed in the environment. Users can interact naturally with the virtual environment through specific devices (e.g., head-mounted displays, joysticks, etc.) (Pastel et al., 2022). In recent years, this technology has played an increasingly important role in studying human behavior in emergency evacuation.

VR technology through 3D modeling to build a realistic simulation environment can be based on the experimental design to regulate the details of the building environment, flame smoke, and the design of the variables in the precise control, to ensure that the experiment has a high degree of ecological validity, for the design of multivariate impact of the experiment has the advantage (Feng et al., 2022). At the same time, the VR experiment has a high degree of safety, in the simulation fire will not cause casualties and property damage; can be repeated many times to reduce the chance of error. In addition, the ability to record the behavioral performance data of users during fire evacuation helps experimenters to identify potential problems and optimize directions after analyzing these data, which provides new insights and ideas for the development of a more reasonable fire evacuation plan (Liu and Liu, 2025). Based on the above advantages, this experiment considers the safety, flexibility, ease of implementation, and relevance of virtual reality technology in human research, and therefore chooses to utilize virtual reality technology to explore pedestrians’ wayfinding decisions in fire evacuation.

### 2.3 Research objectives

In summary, this study aims to provide new insights into pedestrians’ routing behavior during indoor fires from a social impact perspective. To this end, an immersive virtual reality (VR) route-finding decision-making experiment was designed in this study. Different numbers of NPCs (non-game players) were placed on the two evacuation routes in the experimental scenario to simulate the ratio of the number of people at different exits, and different gender ratios (more males than females, fewer males than females, and the same number of males and females) were placed on the two evacuation routes to simulate the distribution of genders choosing different exits, with the aim of exploring the effects of the number of evacuating crowds and the gender-differentiated factors on individual route-finding decisions. In this study, VR was used to obtain real-time data on route choice in a simulated fire emergency.

To summarize, this study contributes to the article on pedestrian fire evacuation by answering the following questions:

1. Is an individual’s route choice influenced by the percentage of people at each exit?
2. Is an individual’s route choice influenced by the percentage of gender at each exit?
3. Does an individual’s route choice depend on the interaction of crowd proportion and gender proportion?

## 3 Materials and methods

### 3.1 Subject

Recruitment of experimental subjects through social networking platforms and other channels for universities in Chengdu, Sichuan Province, with a focus on the student population. The subjects were required to have normal or corrected vision, no color blindness, normal mobility, and no history of heart disease or related illnesses. The experiment required subjects to arrive at the virtual laboratory of Sichuan Normal University at an agreed upon time period to perform the experimental task. *A priori* analysis was conducted through G-power, setting an effect size of 0.25, a significance level (α) of 0.05, and a power of 0.9 to determine a sample size of no less than 141. Finally 162 subjects, 82 males and 80 females, were enrolled and completed the experiment. The age range of the subjects was 19–30 years old, the mean age was 25.21 years old with a standard deviation of 3.53. The experiment was approved by the Ethics Committee.

### 3.2 Apparatus

The experimental scene was modeled and rendered by 3D Studio Max software and then imported into the Unity3D game engine platform. Subjects wore a VR device with a binocular display with a resolution of 1080 (horizontal) × 1200 (vertical) pixels to see the experimental scene and fire smoke in an immersive way and to hear the sound of combustion that simulates the occurrence of a real fire through the headset. During the experiment, participants remained standing and interacted with the IVE by manipulating the handle. Specifically, they manipulated the handle and moved through the IVE, moving forward, backward, left, or right at their speed.

### 3.3 Virtual displays

The experimental scenario is modeled concerning the layout of a college library. Scenario elements include a book display area, student study area, service desk, and two evacuation routes available for evacuation (Layout in both left and right directions), and evacuation indicator signs are placed in both evacuation routes. According to the experimental design, the presence of non-player characters (later replaced by NPCs) was added to the scenario. Before the subjects started to evacuate, the NPCs were unevenly distributed in the study area and the book display area, and carried out normal library study and book reading behaviors, and the NPCs and the subjects did not know each other and did not produce any communication and interaction behaviors. Based on this experiment to investigate the effect of crowd diversion on the subjects’ evacuation route selection, the subjects’ evacuation starting point was farther away from both routes so that when the subjects reached the decision point of route selection, the NPCs made the evacuation route selection before the subjects in different proportions. In addition, there was no significant difference in the distance from the subjects’ starting point to the two evacuation routes, and the tables and chairs in the study area were arranged in an orderly fashion and spaced so that they would not affect the subjects’ evacuation.

### 3.4 Experimental design

To investigate the evacuation route decision-making behavior of pedestrians in an indoor library fire emergency evacuation scenario under the conditions of facing different crowd diversion patterns and different gender distribution ratios of the evacuating crowd. The experiment manipulated two independent variables: crowd distribution pattern (the distribution of the number of NPCs choosing left- or rightward routes for evacuation), and gender distribution pattern (the gender distribution of NPCs choosing left- or rightward evacuation routes). Considering that pedestrians may have a selection preference for evacuation routes in the left or right direction, the direction of evacuation of NPCs was also used as a factor to distinguish between different levels of crowd diversion patterns. In other words, all NPCs choosing evacuation routes to the left belong to different crowd flow patterns than all NPCs choosing evacuation routes to the right. To better distinguish the variability of crowd distribution characteristics in different scenarios, the experiment determined the total number of NPCs in each scenario to be 100.

Crowd flow patterns: The flow proportion of NPCs distributed on the evacuation routes, three levels of flow patterns are designed: 0–10, 5–5, and 7–3. Considering that there may be a directional preference for route selection, symmetric scenarios for crowd flow patterns are added, i.e., five crowd flow patterns: 0–10, 10–0, 5–5, 7–3, and 3–7. For specific examples, 10–0 indicates that all NPCs choose Route 1 for evacuation, while 0–10 indicates that all NPCs choose Route 2 for evacuation. This variable was designed to help explore the effect of the proportion of the number of herd effects on the subjects’ wayfinding decisions.

Gender distribution patterns: The experiment was designed with a hierarchical gender distribution: 7:3, with the specific gender ratios in the later text being male: female, For example, 7:3 means that males accounted for 70% of the total evacuation NPCs who chose the route and females accounted for 30% of the total evacuation NPCs who chose the route; and 3:7 means that males accounted for 30% of the total evacuation NPCs who chose the route and females accounted for 70% of the total evacuation NPCs who chose the route. 70% of the total evacuation NPCs for the route. Considering the design of the flow pattern, the gender distribution ratio then has eight distribution types: 0–7:3, 0–3:7, 7:3–0, 3:7–0, 7:3–7:3, 3:7–3:7, 7:3–3:7, and 3:7–7:3. The variable was designed to explore the effect of the gender ratio of the herd effect on the subjects’ wayfinding decisions.

**FIGURE 1:**
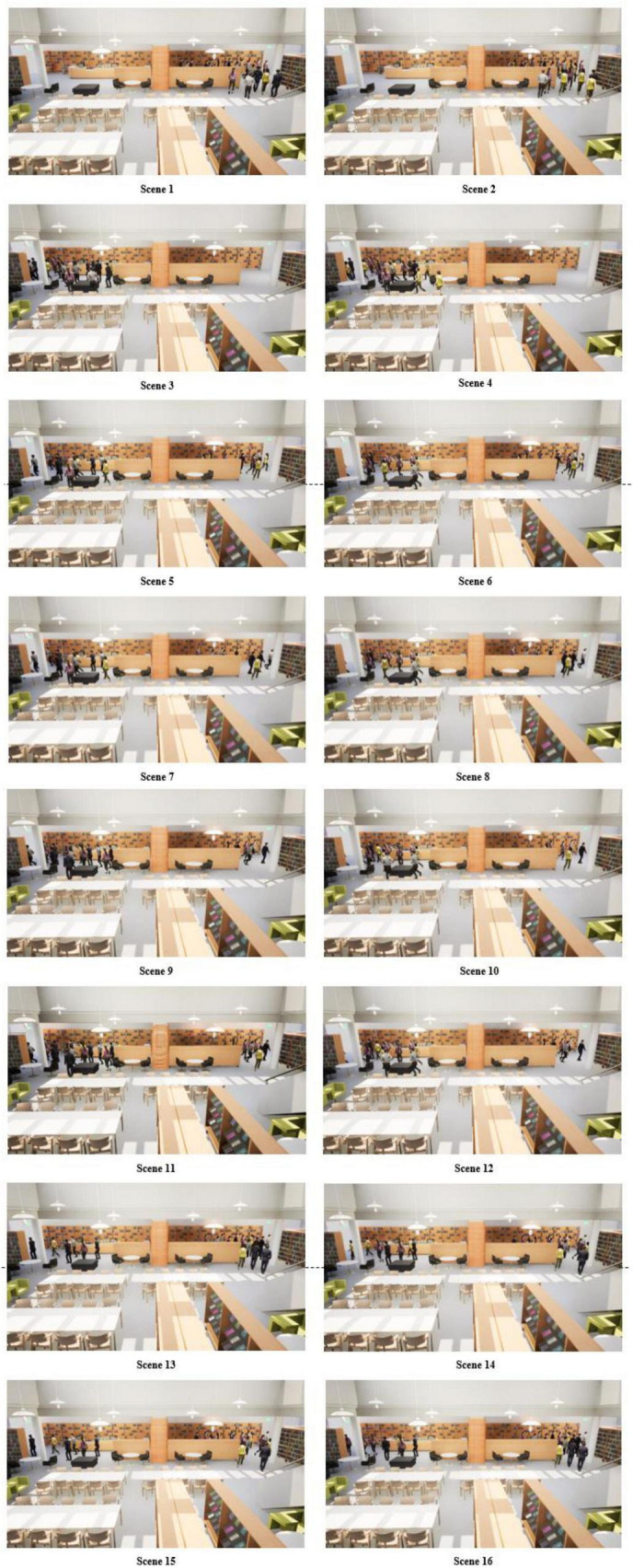
16 VR modeling evacuation scenarios.

According to the design of the combination of experimental variables, there are 16 different experimental scenarios, Table 1 shows the specific combination of the 16 scenarios, and Figure 1 show the modeling scenarios of the 16 scenarios in VR. A total of 162 subjects participated in the route selection of the 16 scenarios in a randomized order, and each scenario took roughly 3 min. The movement speeds of the NPCs in the experimental scenarios ranged from 2.3 m/s to 2.5 m/s.

**TABLE 1:** Design of experimental scene.

Scene	Design	Number of NPCs	
		Route-left	Route-right
1	0 (0:0)–10 (7:3)	Male: 0/female: 0	Male: 70/female: 30
2	0 (0:0)–10 (3:7)	Male: 0/female: 0	Male: 30/female: 70
3	10 (7:3)–0 (0:0)	Male: 70/female: 30	Male: 0/female: 0
4	10 (3:7)–0 (0:0)	Male: 30/female: 70	Male: 0/female: 0
5	5 (7:3)–5 (7:3)	Male: 35/female: 15	Male: 35/female: 15
6	5 (3:7)–5 (3:7)	Male: 15/female: 35	Male: 15/female: 35
7	5 (7:3)–5 (3:7)	Male: 35/female: 15	Male: 15/female: 35
8	5 (3:7)–5 (7:3)	Male: 15/female: 35	Male: 35/female: 15
9	7 (7:3)–3 (7:3)	Male: 49/female: 21	Male: 21/female: 9
10	7 (3:7)–3 (3:7)	Male: 21/female: 49	Male: 9/female: 21
11	7 (7:3)–3 (3:7)	Male: 49/female: 21	Male: 9/female: 21
12	7 (3:7)–3 (7:3)	Male: 21/female: 49	Male: 21/female: 9
13	3 (7:3)–7 (7:3)	Male: 21/female: 9	Male: 49/female: 21
14	3 (3:7)–7 (3:7)	Male: 9/female: 21	Male: 21/female: 49
15	3 (7:3)–7 (3:7)	Male: 21/female: 9	Male: 21/female: 49
16	3 (3:7)–7 (7:3)	Male: 21/female: 9	Male: 49/female: 21

### 3.5 Experimental procedure

1. In the preparatory stage, 162 subjects signed an informed consent form for the experiment and an undertaking that they had no heart disease or related medical history. The experimenter told the subjects that the experiment was to evaluate the effectiveness and experience of the virtual reality technology, and the experimenter promised that if the subjects had any discomfort during the experiment, they could stop the experiment immediately without explaining the reason.
2. Subjects were asked to complete questionnaires that included basic demographic information, the Virtual Reality Syncope Questionnaire (VRSQ), and the Santa Barbara Scale of Orientation (SBSOD).
3. Subjects were asked to read an instruction manual on how to navigate in VR and then were required to put on an HMD and immerse themselves in an empty open space for a brief demonstration to familiarize themselves with the operation of the VR device and the immersive feeling of the virtual environment.
4. The formal experiment began, the subjects entered the virtual experimental scene, according to the requirements of the experimenter to reach the designated location and look for the corresponding book, the subjects arrived at the starting point 5 s, the fire alarm sounded, the screen displayed “please quickly evacuate,” the subjects began to carry out the evacuation. After successfully reaching the exit, the experiment ends.
5. At the end of the experiment, the subjects filled out the VRSQ questionnaires again, and the experimenter explained the purpose of the experiment to the subjects and thanked them for their active cooperation.

## 4 Results

### 4.1 Data collection and analysis

The experiment explored the route choice decisions of pedestrians in a high-rise library fire outbreak scenario. Two evacuation routes were designed for a controlled experiment, and 16 specific evacuation scenarios were constituted by varying the proportion of NPC traffic on the evacuation routes, as well as the proportion of NPC men and women. Each subject performed 16 evacuation route choices in the VR library scenarios, and the test order of the 16 scenarios was randomized. Participant behavior was assessed by collecting evacuation routes, and a total of 2,592 route choice data were collected.

The steps for analyzing the experimental data are stated separately according to the previously mentioned research questions. First, the chi-square test was utilized to investigate the effect of the proportion of the number of NPCs exiting on individual route choice and the effect of the proportion of the gender of the NPCs exiting on individual route choice, respectively. Next, a generalized linear model was developed to explore the effect of the interaction between the proportion of NPCs in numbers exported and the proportion of gender on individual route choice. The significance level for data analysis was set at 0.05 and SPSS 27 software was used to complete all analyses.

### 4.2 The effect of the proportion of people who exit on individual route choice

**FIGURE 2:**
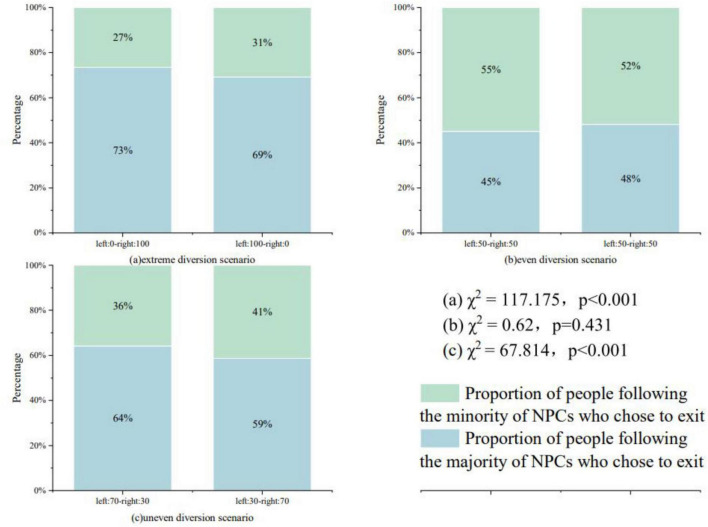
**(a)** Route selection for extreme crowd diversion scenarios. **(b)** Route selection for even crowd diversion scenarios. **(c)** Route selection uneven crowd diversion scenarios.

Figure 2 demonstrates the proportion of evacuation routes chosen by the subjects under different distribution ratios of the number of NPCs. (a) The setup scenarios with extreme distribution ratios of the crowd include Scenario 1-Scenario 4. left: 0-right: 100 indicates the scenario in which all 100 NPCs chose the road to the right for evacuation and no NPCs chose the road to the left for evacuation. Where left: 0-right: 100 merges the route choice data for scenarios 1–2 and left: 100-right: 0 merges the subject data for scenarios 3–4. As can be seen in the figure, when all NPCs chose the road to the right, 73% (238) of the subjects chose to follow the NPCs; when all NPCs chose the road to the left, 69% (224) of the subjects chose to follow the NPCs, the chi-square results indicate that the extreme diversion ratio significantly affects the pedestrian’s route choice (χ^2^ = 117.175, *p* < 0.001),pedestrians chose to follow the majority of the people for evacuation (73% > 27%, 69% > 31%).

(b) The setup scenarios for the uniform distribution ratio of the crowd include Scenario 5–Scenario 8, and the number of NPCs choosing the two evacuation routes is consistent at 50. Thus the data from randomly merged scenarios 5–6 are compared to the data from merged scenarios 7–8, and the same color in Figure 2b indicates the proportion of subjects choosing the same route. The chi-square test did not find a significant difference (χ^2^ = 0.62, *p* = 0.431 > 0.05), which illustrates that there is no directional preference (preference to the left or to the right) in the pedestrians’ route choice.

(c) The setup scenarios for the uneven distribution ratio of the crowd include Scenario 9–Scenario 16, left: 70-right: 30 indicates that 70 NPCs chose to evacuate to the left of the road, and 30 NPCs chose to evacuate to the right of the road. Where left: 70-right: 30 data are from scenarios 9–12 and left: 30-right: 70 data are from scenarios 13–16. When the majority of NPCs chose to evacuate to the left, 64% (416) of the subjects chose to follow; when the majority of NPCs chose to evacuate to the right, 59% (380) of the subjects chose to follow. The chi-square test results proved that the uneven diversion pattern (χ^2^ = 67.814, *p* < 0.001), pedestrians tend to choose to follow the majority for evacuation (64% > 36%, 59% > 41%).

### 4.3 The effect of the proportion of gender at the exit on individual route choice

**FIGURE 3:**
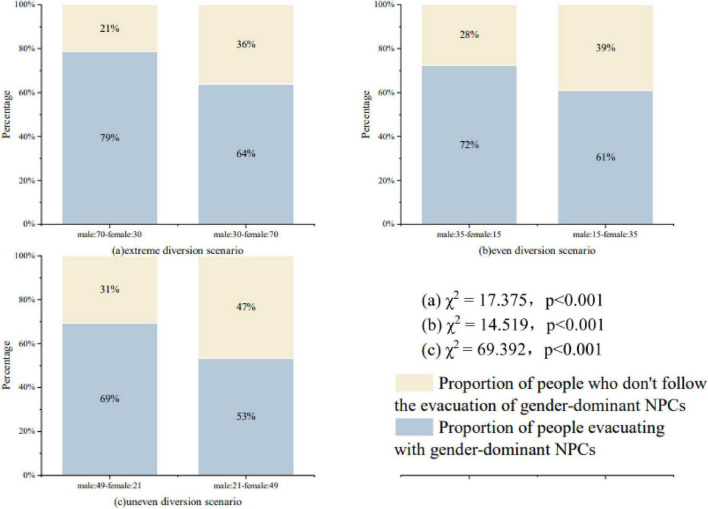
**(a)** Route selection for different gender distributions in extreme crowd diversion scenarios. **(b)** Route selection for different gender distributions in even crowd diversion scenarios. **(c)** Route selection for different gender distributions in uneven crowd diversion scenarios.

Figure 3 shows the proportion of evacuation routes chosen by subjects with different gender distribution ratios of NPCs. (a) In the extreme diversion scenario, all NPCs choose a certain evacuation route for evacuation, but different gender ratios are set. male: 70-female: 30 indicates that when all NPCs choose to evacuate to the left or to the right, there are 70 male NPCs, and 30 female NPCs; where male: 70-female: 30 combines the route choice data for Scenario 1 and Scenario 3, and male: 30-female: 70 combines the route choice data for Scenario 2 and Scenario 4. When males are in the majority, 79% (255) subjects choose to follow the evacuation; when females are in the majority, 64% (207) subjects choose to follow the evacuation. When males were in the majority, 79% (255) of the subjects chose to follow the evacuation; when females were in the majority, 64% (207) of the subjects chose to follow. After a chi-square test (χ^2^ = 17.375, *p* < 0.001), it illustrates that pedestrians tend to follow the evacuation of subjects with a greater proportion of males in extreme diversion scenarios (79% > 64%).

(b) Uneven diversion scenario, male: 35-female: 15 denotes the evacuation routes chosen by 35 male NPCs and 15 female NPCs. male: 35-female: 15 male NPCs are in the majority to follow the crowd evacuation data with a gender advantage comes from the evacuation routes to the left and to the right of scenario 5, the evacuation routes to the left of scenario 7 Male: 15-female: 35 indicates the evacuation routes chosen by 15 male NPCs and 35 female NPCs, and the evacuation routes chosen by the majority of male: 15-female: 35 female NPCs following the crowd with gender advantage are derived from the evacuation routes to the left and to the right of scenario 6, the evacuation routes to the right of scenario 7, the evacuation routes to the right of scenario 8, and the evacuation routes to the right of scenario 8, and the evacuation routes to the right of scenario 8, and the evacuation routes to the right of scenario 8, and the evacuation routes to the right of scenario 8, respectively. evacuation routes, and leftward evacuation route choices from Scene 8. The chi-square results (χ^2^ = 14.519, *p* < 0.001) found a significant effect of gender ratio on pedestrian route choice, with pedestrians tending to follow the route with a greater number of males for evacuation when the number of NPCs route choices was the same in both directions (72% > 61%).

(c) Uneven diversion scenario is the existence of a certain gender NPCs dominate in a specific direction. male: 49-female: 21 indicates that the gender distribution of NPCs choosing a certain evacuation route for evacuation is 49 male NPCs and 21 female NPCs. where male: 49-female: 21 merged the subject route choice data for scenarios 9, 11, 13, and 16, and male: 21-female: 49 merged the subject route choice data for scenarios 10, 12, 14, and 15. As can be seen in the figure, 69% (450) of the subjects tended to follow when males dominated the number of genders in a particular evacuation route, while 53% (346) of the subjects tended to follow when females dominated the number of genders in a particular evacuation route. The chi-square results showed a significant difference in pedestrians’ evacuation route choices (χ^2^ = 69.392, *p* < 0.001), proving that pedestrians were more inclined to follow males in evacuation (69% > 53%).

### 4.4 The effect of the interaction between the proportion of people exported and the proportion of gender on individual route choice

The generalized linear model can be used to analyze the dichotomous dependent variable, which was used to analyze the existence of interaction of independent variables. The omnibus test of the model has *P* < 0.001, so the original hypothesis is rejected and the model as a whole is considered to be significant, i.e., the independent variables have a significant explanatory power for the dependent variable. To investigate whether the uneven distribution of crowd size produces a herding effect on pedestrians, the crowd distribution patterns were coded as 0 (even crowd distribution), 1 (extreme crowd distribution), and 2 (uneven crowd distribution). The contrast terms were 0, i.e., 1 vs. 0 and 2 vs. 0. For the gender distribution ratios coded as 1 (majority number of male NPCs) and 2 (majority number of female NPCs), this was coded to explore whether pedestrians were more inclined to follow the male/female group for evacuation.

Table 2 displays the generalized linear model effect test values, and the results show that the population distribution patterns (0–1: Waldχ^2^ = 9.644, *P* = 0.002; 0–2: Waldχ^2^ = 6.363, *P* = 0.012) and gender distribution patterns (1–2: Waldχ^2^ = 34.821, *P* < 0.001) had a main effect on pedestrian route choice, but the interaction effect was not significant. Possible reasons why the interaction effect was not significant will be explored in the discussion section.

**TABLE 2:** Generalized linear model results.

Factors	B	SE	Wald χ^2^	df	*p*-value	Exp (B)	95% CI	
							Lower	Upper
**Comparison item: even crowd patter (0)**								
0–1	−0.435	0.1399	9.644	1	0.002**	0.648	0.492	0.852
0–2	−0.307	0.1218	6.363	1	0.012*	0.735	0.579	0.934
**Comparison item: majority number of male NPCs (1)**								
1–2	−0.685	0.1161	34.821	1	< 0.001**	0.504	0.402	0.633
**Interaction effect**								
(crowd = 1*gender = 1) -crowd = 1*gender = 2	0.163	0.1801	0.815	1	0.367	1.176	0.827	1.674
(crowd = 2*gender = 1) -crowd = 2*gender = 2	−0.052	0.2128	0.059	1	0.808	0.95	0.626	1.441

*p < 0.05;

**p < 0.01. The explanation of specific values is explained in the text section of “4.4 The effect of the interaction between the proportion of people exported and the proportion of gender on individual route choice.”

### 4.5 Validity assessment

In this study, two standardized instruments, the Virtual Reality Stun Scale (VRSQ) and the Sense of Spatial Orientation Scale (SBSOD), were used to assess the validity of the virtual experiment. The potential interference of the virtual reality environment on the experimental results was first analyzed by the VRSQ, which contains 9 entries on a Likert-type scale from 0 (not at all) to 3 (very much). The reliability test of the scale showed a Cronbach’s alpha coefficient of 0.91, indicating a high degree of internal consistency of the measurement instrument. Statistical analysis based on the Wilcoxon signed rank test showed that the subjects’ mean VRSQ scores before and after the experiment did not show significant changes (1.49 vs. 1.52), and the between-group comparisons of each sub-index were not statistically significant (*p* > 0.05), proving that the VR environmental factors had a limited effect on the experimental results. For the assessment of spatial cognitive abilities, the study pretested and analyzed subjects’ spatial orientation abilities using the SBSOD scale, which consists of 15 items on a Likert-type scale of 0 (not at all compliant) to 7 (fully compliant). The reliability test of the scale showed a Cronbach’s alpha coefficient of 0.89, indicating a high degree of internal consistency of the measurement instrument. Comparison by Mann–Whitney U test revealed that the difference in the mean of the total SBSOD scores of different subject groups did not reach a significant level (54.87 vs. 56.29), which illustrated that there was no significant difference in spatial cognitive ability among subjects. Taken together, the results of the data from the two assessment tools together validated the internal validity of the present study, confirming the reliability and validity of the experimental findings.

## 5 Discussion

Experiments on pedestrian routing in an immersive virtual library fire emergency scenario using VR technology have increased the research on pedestrian behavior in emergency fire evacuation and contributed to the understanding of pedestrian routing decisions. A large number of previous studies have investigated the herd effect in fire evacuation, but few studies have comprehensively considered the effect of different crowd mobility ratios on pedestrians’ tendency to follow, and few studies have considered whether the gender ratio of the crowd affects pedestrians’ following behavior. Our study attempts to explore the above issues.

In the experiment, when the pedestrians hear the fire alarm ringing, they immediately start to evacuate, and when they reach the route decision point, the pedestrians obtain the signal of safe evacuation by sensing the evacuation information of the surrounding environment. It has been pointed out that the sensory information utilized by pedestrians during evacuation is divided into dynamic and static information, and the static information mainly includes emergency lighting, distance to exits, evacuation signs, corridors, staircases, elevators, and alarms; and the dynamic information includes the behaviors of the surrounding people, and the spread of the fire and the smoke generated (Lovreglio et al., 2016b; Gao et al., 2023). The static environmental information of the two paths in this experimental design remained consistent, both had evacuation instruction signs, and the subjects could observe the presence of fire smoke in both evacuation paths, and the experimental NPCs were triaged according to the scenario design and evacuated according to the predetermined paths. The evacuation of crowds serves as a dynamic factor that plays a guiding role in the experiment.

The study found that pedestrians tend to follow the route chosen by a larger majority of the crowd for evacuation, especially in the extreme scenario where the NPC crowd concentrates on choosing the same evacuation path, more than 70% of the subjects still choose to follow the crowd for evacuation (Figure 2). This finding is consistent with the conclusions of most herd effect studies. In terms of information processing, crowd information is the most easily captured dynamic information that helps pedestrians make emergency decisions, the direction of large crowd evacuation demonstrates the sharing of safety information, and subjects believe that the path chosen by the majority of the crowd can reach the exit faster and away from the fire when making emergency decisions. Considering from an emotional perspective, pedestrians crave group support and protection, following the majority of the crowd to evacuate helps to share the perceived risk, reduces anxiety to some extent, and provides psychological comfort.

According to Figure 3, the results show that differences in gender distribution significantly affect pedestrians’ route choices. When the NPC crowd concentrates on choosing the same direction for evacuation, pedestrians are more likely to follow the crowd flow with a higher proportion of males to evacuate, while the number of subjects who follow the crowd flow with a higher proportion of females to evacuate is significantly lower. Based on the analysis from the perspective of role position theory, men are traditionally viewed as showing more strength, possessing the ability to make rational decisions in emergencies, and being able to take the lead in responding to crises; whereas women are portrayed as being more dependent, showing more empathy, and tending to cooperate with others (Rosenthal et al., 2012); and are more likely to show panic in emergencies, thus resulting in increased stress reducing the possibility of rational decision-making (Haghani and Sarvi, 2019a; Haghani and Sarvi, 2019b). On the other hand, spatial cognition studies have shown that males are more dominant for spatial wayfinding, with a higher degree of route mastery and judgment compared to females (Zhu et al., 2024). Males are bolder, engage in risky behaviors, and are likely to be the first to make route choices, and those who are the first to decide in an emergency have a significant impact on those who make decisions with those who follow.

According to the results of Table 2, no significant interaction effect was found between the proportion of crowd distribution and the proportion of gender distribution on the choice of pedestrian evacuation routes, but according to the statistical results of route choice, when the majority of the crowd evacuation direction is the same as the majority of the male evacuation direction, the proportion of subjects’ choice for the route will be greater (79% > 64%). The reason that the experiment did not find a significant effect may be that the sample size was insufficient, resulting in insufficient statistical test power; or the subjects had fewer available evacuation routes, making the data less variable and masking the interaction between the independent variables. In the future, the level of independent variables and the complexity of the experimental scenarios can be further enriched to explore the interaction between the proportion of population distribution and the proportion of gender distribution.

Our findings have important implications for evacuation training and emergency management. When evacuating indoor environments in unfamiliar scenarios, the evacuation direction of the majority of the crowd significantly increases the pedestrian’s choice of that route, which is highly likely to lead to serious congestion and stampede accidents. Therefore, it is necessary to emphasize the avoidance of blindly following the crowd in evacuation decision-making in evacuation training to strengthen pedestrians’ awareness of the seriousness of congestion and stampede accidents. In emergency management, it is also necessary to take specific measures to reduce blind following behavior, such as increasing the visibility of evacuation signs to achieve the effect of diverting people from evacuation. Pedestrians are more likely to follow men in route selection, and considering the role of men, it is recommended that training for staff in indoor scenarios pay particular attention to reinforcing the leadership role of men in directing evacuation.

Of course, our study still has limitations: (1) Although a large number of studies have proved the ecological validity of immersive virtual reality technology in the field of emergency evacuation, the current technology is based on visual stimulation and auditory stimulation, and future technology is expected to add stimulation to the senses of smell and touch, which will further enhance the subjects’ sense of immersion in reality and the validity of the results. (2) The subjects in this experiment were mainly from the student group, and the group sample was The group sample is not representative enough, and a separate study should be considered for the elderly and other groups with basic mobility deficiencies. (3) The experimental scenario is a simple indoor library evacuation scenario, and pedestrians in complex indoor spatial layouts are confronted with more information and more difficulty in evacuating, so it is necessary to conduct an in-depth study of evacuation in complex indoor scenarios. (4) The proportion of the NPC crowd diversion ratio and the gender distribution ratio of the evacuation route need to be further refined. (5) The subjects in this study make decisions as independent individuals, and the study is aimed at individual evacuation behavior, while in the evacuation of the actual scene, there are often familiar groups around, and future research needs to further consider the group evacuation behavior.

## Data Availability

The raw data supporting the conclusions of this article will be made available by the authors, without undue reservation.
